# Supplementary material for: Genome-Wide Identification MIKC-Type MADS-Box Gene Family and Their Roles during Development of Floral Buds in Wheel Wingnut (Cyclocarya paliurus)
Source: Int J Mol Sci. 2021 Sep 19;22(18):10128. doi: 10.3390/ijms221810128 (PMC8471257; doi:10.3390/ijms221810128)
Supplement: Supplementary file 1 [file ijms-22-10128-s001.zip › Supplemental Fig. S1.pdf]

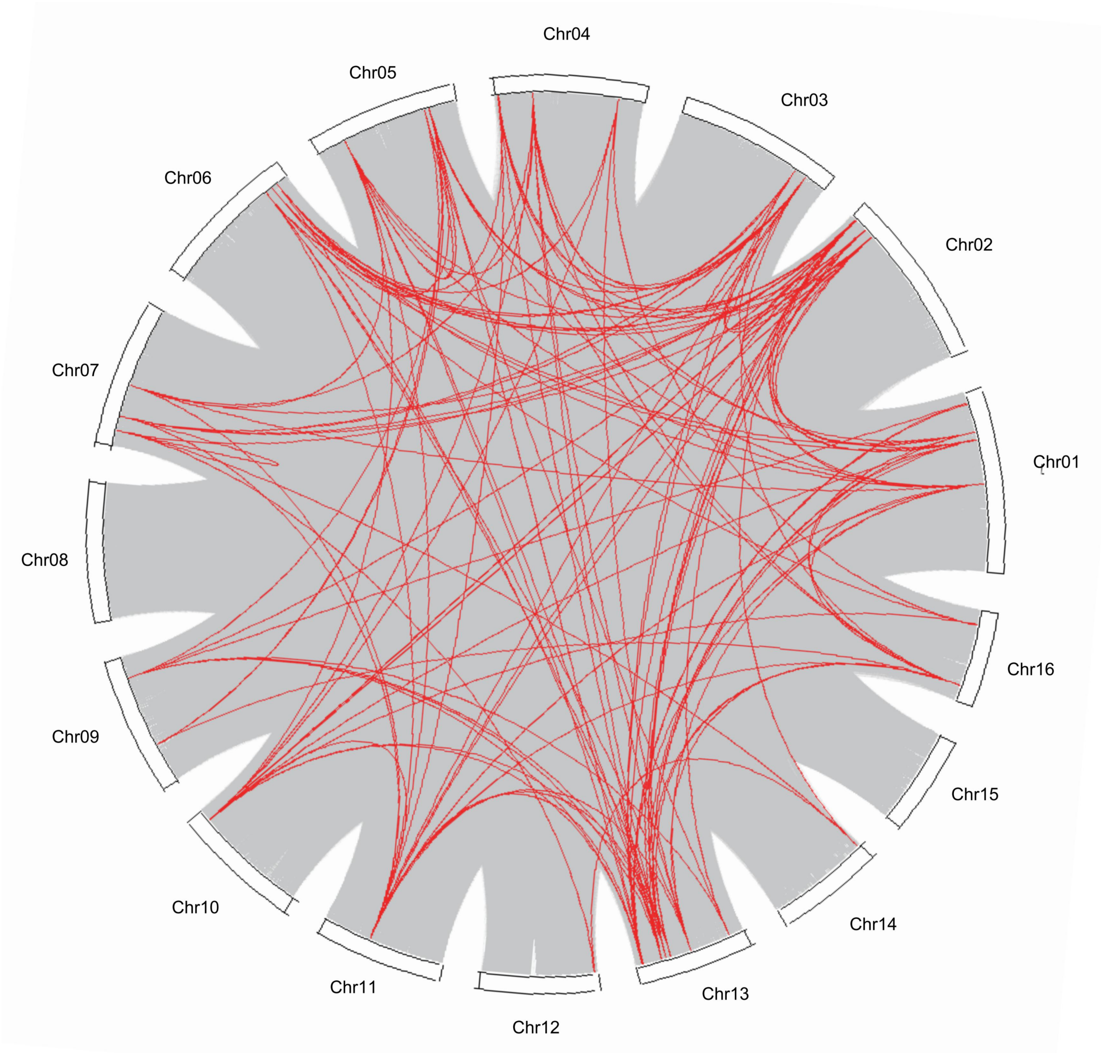

Figure S1. Synteny analysis of apple MIKC-type MADS-box genes. Red lines indicate duplicated MIKC-type MADS-box gene pairs. The chromosome number is indicated at the bottom of each chromosome.
