# Supplementary material for: Genome-Wide Identification MIKC-Type MADS-Box Gene Family and Their Roles during Development of Floral Buds in Wheel Wingnut (Cyclocarya paliurus)
Source: Int J Mol Sci. 2021 Sep 19;22(18):10128. doi: 10.3390/ijms221810128 (PMC8471257; doi:10.3390/ijms221810128)
Supplement: Supplementary file 1 [file ijms-22-10128-s001.zip › Supplemental Fig. S2.pdf]

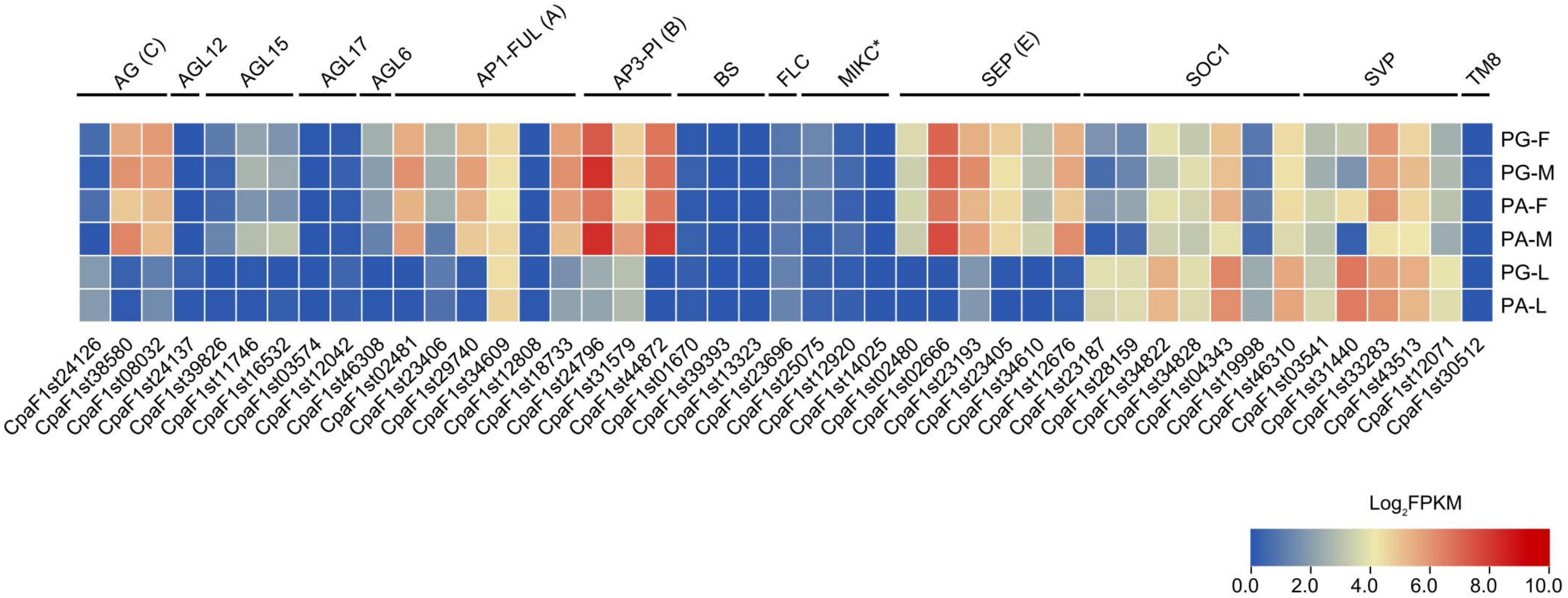

Figure S2. Heatmap showing the expression patterns of 45 MIKC-type MADS-box genes among PG and PA floral buds. (PG-F) Female, (PA-F) Female, (PG-M) Male, (PA-M) Male flower buds.
