# Supplementary material for: Genome-Wide Identification MIKC-Type MADS-Box Gene Family and Their Roles during Development of Floral Buds in Wheel Wingnut (Cyclocarya paliurus)
Source: Int J Mol Sci. 2021 Sep 19;22(18):10128. doi: 10.3390/ijms221810128 (PMC8471257; doi:10.3390/ijms221810128)
Supplement: Supplementary file 1 [file ijms-22-10128-s001.zip › Supplemental Tab. S1.pdf]

Table S1. Analysis of segmental duplication events of MADS-box gene pairs in *C. paliurus.*

Locus1	Chromosome localization	Locus2	Chromosome localization
CpaF1st01670	Chr1:22512748-22522152	CpaF1st24126	Chr2:42674312-42685121
CpaF1st01670	Chr1:22512748-22522152	CpaF1st25075	Chr3:8607733-8610986
CpaF1st01670	Chr1:22512748-22522152	CpaF1st31440	Chr5:7130147-7137608
CpaF1st01670	Chr1:22512748-22522152	CpaF1st33283	Chr5:30392209-30399648
CpaF1st01670	Chr1:22512748-22522152	CpaF1st38580	Chr5:30392209-30399648
CpaF1st01670	Chr1:22512748-22522152	CpaF1st08032	Chr11:12616678-12625690
CpaF1st01670	Chr1:22512748-22522152	CpaF1st12042	Chr11:12616678-12625690
CpaF1st01670	Chr1:22512748-22522152	CpaF1st13323	Chr13:14729791-14740772
CpaF1st01670	Chr1:22512748-22522152	CpaF1st18733	Chr13:14729791-14740772
CpaF1st02480	Chr1:34029459-34037773	CpaF1st23405	Chr2:36737147-36746074
CpaF1st02480	Chr1:34029459-34037773	CpaF1st23696	Chr2:39302712-39326595
CpaF1st02480	Chr1:34029459-34037773	CpaF1st29740	Chr4:28769871-28783403
CpaF1st02480	Chr1:34029459-34037773	CpaF1st34609	Chr6:4893230-4907152
CpaF1st02480	Chr1:34029459-34037773	CpaF1st04343	Chr10:3501933-3522124
CpaF1st02480	Chr1:34029459-34037773	CpaF1st12808	Chr13:8140813-8160527
CpaF1st02480	Chr1:34029459-34037773	CpaF1st18733	Chr16:3824143-3832006
CpaF1st02666	Chr1:36013106-36019785	CpaF1st23696	Chr2:39302712-39326595
CpaF1st02666	Chr1:36013106-36019785	CpaF1st24137	Chr2:42770150-42773456
CpaF1st02666	Chr1:36013106-36019785	CpaF1st30512	Chr4:37577074-37585817
CpaF1st02666	Chr1:36013106-36019785	CpaF1st12676	Chr13:6960414-6973803
CpaF1st02666	Chr1:36013106-36019785	CpaF1st12808	Chr13:8140813-8160527
CpaF1st02666	Chr1:36013106-36019785	CpaF1st18733	Chr16:3824143-3832006
CpaF1st02666	Chr1:36013106-36019785	CpaF1st46308	tig00001919:5147437-5153312
CpaF1st03541	Chr1:43429845-43432936	CpaF1st12071	Chr13:1904102-1910372
CpaF1st03574	Chr1:43721431-43725962	CpaF1st43513	Chr9:5780448-5787523
CpaF1st03574	Chr1:43721431-43725962	CpaF1st12042	Chr13:1686053-1691438
CpaF1st04343	Chr10:3501933-3522124	CpaF1st23405	Chr2:36737147-36746074
CpaF1st04343	Chr10:3501933-3522124	CpaF1st24126	Chr2:42674312-42685121
CpaF1st04343	Chr10:3501933-3522124	CpaF1st24137	Chr2:42770150-42773456
CpaF1st04343	Chr10:3501933-3522124	CpaF1st28159	Chr4:6573859-6580422
CpaF1st04343	Chr10:3501933-3522124	CpaF1st29740	Chr4:28769871-28783403
CpaF1st04343	Chr10:3501933-3522124	CpaF1st33283	Chr5:30392209-30399648
CpaF1st04343	Chr10:3501933-3522124	CpaF1st08032	Chr11:12616678-12625690
CpaF1st04343	Chr10:3501933-3522124	CpaF1st12042	Chr13:1686053-1691438
CpaF1st04343	Chr10:3501933-3522124	CpaF1st12808	Chr13:8140813-8160527
CpaF1st04343	Chr10:3501933-3522124	CpaF1st19998	Chr16:19997527-20003350
CpaF1st04343	Chr10:3501933-3522124	CpaF1st46308	tig00001919:5147437-5153312
CpaF1st08032	Chr11:12616678-12625690	CpaF1st23405	Chr2:36737147-36746074
CpaF1st08032	Chr11:12616678-12625690	CpaF1st24126	Chr2:42674312-42685121
CpaF1st08032	Chr11:12616678-12625690	CpaF1st24796	Chr3:5479964-5482602
CpaF1st08032	Chr11:12616678-12625690	CpaF1st30512	Chr4:37577074-37585817
CpaF1st08032	Chr11:12616678-12625690	CpaF1st33283	Chr5:30392209-30399648

CpaF1st08032	Chr11:12616678-12625690	CpaF1st34822	Chr6:6894591-6915492
CpaF1st08032	Chr11:12616678-12625690	CpaF1st38580	Chr7:21011822-21022154
CpaF1st08032	Chr11:12616678-12625690	CpaF1st39393	Chr7:29269125-29273783
CpaF1st08032	Chr11:12616678-12625690	CpaF1st12042	Chr13:1686053-1691438
CpaF1st08032	Chr11:12616678-12625690	CpaF1st12920	Chr13:9350856-9357523
CpaF1st08032	Chr11:12616678-12625690	CpaF1st13323	Chr13:14729791-14740772
CpaF1st08032	Chr11:12616678-12625690	CpaF1st18733	Chr16:3824143-3832006
CpaF1st11746	Chr12:29355993-29360302	CpaF1st39826	Chr7:33119145-33121865
CpaF1st12042	Chr13:1686053-1691438	CpaF1st24137	Chr2:42770150-42773456
CpaF1st12042	Chr13:1686053-1691438	CpaF1st24796	Chr3:5479964-5482602
CpaF1st12042	Chr13:1686053-1691438	CpaF1st25075	Chr3:8607733-8610986
CpaF1st12071	Chr13:1904102-1910372	CpaF1st31440	Chr5:7130147-7137608
CpaF1st12071	Chr13:1904102-1910372	CpaF1st33283	Chr5:30392209-30399648
CpaF1st12071	Chr13:1904102-1910372	CpaF1st43513	Chr9:5780448-5787523
CpaF1st12676	Chr13:1904102-1910372	CpaF1st23696	Chr2:39302712-39326595
CpaF1st12676	Chr13:1904102-1910372	CpaF1st30512	Chr4:37577074-37585817
CpaF1st12676	Chr13:1904102-1910372	CpaF1st33283	Chr5:30392209-30399648
CpaF1st12676	Chr13:1904102-1910372	CpaF1st43513	Chr9:5780448-5787523
CpaF1st12676	Chr13:1904102-1910372	CpaF1st12808	Chr13:8140813-8160527
CpaF1st12808	Chr13:8140813-8160527	CpaF1st23405	Chr2:36737147-36746074
CpaF1st12808	Chr13:8140813-8160527	CpaF1st23696	Chr2:39302712-39326595
CpaF1st12808	Chr13:8140813-8160527	CpaF1st29740	Chr4:28769871-28783403
CpaF1st12808	Chr13:8140813-8160527	CpaF1st18733	Chr16:3824143-3832006
CpaF1st12808	Chr13:8140813-8160527	CpaF1st46308	tig00001919:5147437-5153312
CpaF1st12920	Chr13:9350856-9357523	CpaF1st24137	Chr2:42770150-42773456
CpaF1st12920	Chr13:9350856-9357523	CpaF1st34822	Chr6:6894591-6915492
CpaF1st12920	Chr13:9350856-9357523	CpaF1st43513	Chr9:5780448-5787523
CpaF1st12920	Chr13:9350856-9357523	CpaF1st18733	Chr16:3824143-3832006
CpaF1st13323	Chr13:14729791-14740772	CpaF1st28159	Chr4:6573859-6580422
CpaF1st13323	Chr13:14729791-14740772	CpaF1st29740	Chr4:28769871-28783403
CpaF1st13323	Chr13:14729791-14740772	CpaF1st34822	Chr6:6894591-6915492
CpaF1st13323	Chr13:14729791-14740772	CpaF1st18733	Chr16:3824143-3832006
CpaF1st13323	Chr13:14729791-14740772	CpaF1st46308	tig00001919:5147437-5153312
CpaF1st14025	Chr13:25286688-25289400	CpaF1st24137	Chr2:42770150-42773456
CpaF1st14025	Chr13:25286688-25289400	CpaF1st31579	Chr5:8498499-8507170
CpaF1st14025	Chr13:25286688-25289400	CpaF1st43513	Chr9:5780448-5787523
CpaF1st18733	Chr16:3824143-3832006	CpaF1st24796	Chr3:5479964-5482602
CpaF1st18733	Chr16:3824143-3832006	CpaF1st29740	Chr4:28769871-28783403
CpaF1st18733	Chr16:3824143-3832006	CpaF1st30512	Chr4:37577074-37585817
CpaF1st18733	Chr16:3824143-3832006	CpaF1st44872	Chr9:24368385-24370857
CpaF1st19998	Chr16:3824143-3832006	CpaF1st28159	Chr4:6573859-6580422
CpaF1st19998	Chr16:3824143-3832006	CpaF1st34822	Chr6:6894591-6915492
CpaF1st19998	Chr16:3824143-3832006	CpaF1st46308	tig00001919:5147437-5153312
CpaF1st23405	Chr2:36737147-36746074	CpaF1st24796	Chr3:5479964-5482602

CpaF1st23405	Chr2:36737147-36746074	CpaF1st31440	Chr5:7130147-7137608
CpaF1st23405	Chr2:36737147-36746074	CpaF1st34609	Chr6:4893230-4907152
CpaF1st23405	Chr2:36737147-36746074	CpaF1st39393	Chr7:29269125-29273783
CpaF1st23696	Chr2:39302712-39326595	CpaF1st24137	Chr2:42770150-42773456
CpaF1st23696	Chr2:39302712-39326595	CpaF1st24796	Chr3:5479964-5482602
CpaF1st23696	Chr2:39302712-39326595	CpaF1st30512	Chr4:37577074-37585817
CpaF1st24126	Chr2:42674312-42685121	CpaF1st31440	Chr5:7130147-7137608
CpaF1st24126	Chr2:42674312-42685121	CpaF1st33283	Chr5:30392209-30399648
CpaF1st24126	Chr2:42674312-42685121	CpaF1st38580	Chr7:21011822-21022154
CpaF1st24126	Chr2:42674312-42685121	CpaF1st46308	tig00001919:5147437-5153312
CpaF1st24137	Chr2:42674312-42685121	CpaF1st31440	Chr5:7130147-7137608
CpaF1st24137	Chr2:42674312-42685121	CpaF1st34609	Chr6:4893230-4907152
CpaF1st24137	Chr2:42674312-42685121	CpaF1st34822	Chr6:6894591-6915492
CpaF1st24137	Chr2:42674312-42685121	CpaF1st39393	Chr7:29269125-29273783
CpaF1st24137	Chr2:42674312-42685121	CpaF1st39826	Chr7:33119145-33121865
CpaF1st24137	Chr2:42674312-42685121	CpaF1st43513	Chr9:5780448-5787523
CpaF1st24137	Chr2:42674312-42685121	CpaF1st46308	tig00001919:5147437-5153312
CpaF1st24796	Chr3:5479964-5482602	CpaF1st25075	Chr3:8607733-8610986
CpaF1st24796	Chr3:5479964-5482602	CpaF1st29740	Chr4:28769871-28783403
CpaF1st24796	Chr3:5479964-5482602	CpaF1st30512	Chr4:37577074-37585817
CpaF1st24796	Chr3:5479964-5482602	CpaF1st31440	Chr5:7130147-7137608
CpaF1st24796	Chr3:5479964-5482602	CpaF1st34609	Chr6:4893230-4907152
CpaF1st24796	Chr3:5479964-5482602	CpaF1st34822	Chr6:6894591-6915492
CpaF1st24796	Chr3:5479964-5482602	CpaF1st39393	Chr7:29269125-29273783
CpaF1st25075	Chr3:8607733-8610986	CpaF1st29740	Chr4:28769871-28783403
CpaF1st25075	Chr3:8607733-8610986	CpaF1st39393	Chr7:29269125-29273783
CpaF1st28159	Chr4:6573859-6580422	CpaF1st46308	tig00001919:5147437-5153312
CpaF1st29740	Chr4:28769871-28783403	CpaF1st33283	Chr5:30392209-30399648
CpaF1st29740	Chr4:28769871-28783403	CpaF1st38580	Chr7:21011822-21022154
CpaF1st31440	Chr4:28769871-28783403	CpaF1st33283	Chr5:30392209-30399648
CpaF1st31440	Chr4:28769871-28783403	CpaF1st34609	Chr6:4893230-4907152
CpaF1st31440	Chr4:28769871-28783403	CpaF1st43513	Chr9:5780448-5787523
CpaF1st31440	Chr4:28769871-28783403	CpaF1st44872	Chr9:24368385-24370857
CpaF1st31440	Chr4:28769871-28783403	CpaF1st46308	tig00001919:5147437-5153312
CpaF1st31579	Chr4:28769871-28783403	CpaF1st33283	Chr5:30392209-30399648
CpaF1st31579	Chr4:28769871-28783403	CpaF1st44872	Chr9:24368385-24370857
CpaF1st33283	Chr5:30392209-30399648	CpaF1st38580	Chr7:21011822-21022154
CpaF1st34822	Chr6:6894591-6915492	CpaF1st46308	tig00001919:5147437-5153312
CpaF1st34828	Chr6:6894591-6915492	CpaF1st46310	tig00001919:5164160-5180657
CpaF1st39393	Chr7:29269125-29273783	CpaF1st39826	Chr7:33119145-33121865
CpaF1st43513	Chr9:5780448-5787523	CpaF1st46308	tig00001919:5147437-5153312
