# Supplementary material for: Genome-Wide Identification MIKC-Type MADS-Box Gene Family and Their Roles during Development of Floral Buds in Wheel Wingnut (Cyclocarya paliurus)
Source: Int J Mol Sci. 2021 Sep 19;22(18):10128. doi: 10.3390/ijms221810128 (PMC8471257; doi:10.3390/ijms221810128)
Supplement: Supplementary file 1 [file ijms-22-10128-s001.zip › Supplemental Tab. S2.pdf]

Table S2. Gene list of of the MIKC Group of the *Arabidopsis* MADS-Box Gene Family.

Gene name	Gene ID
AGL7	AT1G69120
AGL8	AT5G60910
AGL10	AT1G26310
AP3	AT3G54340
PI	AT5G64070
AG	AT4G18960
AGL1	AT3G58780
AGL5	AT2G42830
AG11	AT4G09960
SEP1	AT5G15800
SEP2	AT3G02310
SEP3	AT1G24260
AGL19	AT4G22950
AGL14	AT4G11880
AGL20	AT2G45660
AGL42	AT5G62165
AGL71	AT5G51870
AGL72	AT5G51860
AGL24	AT4G24540
SVP	AT2G22540
AGL6	AT2G45650
AGL12	AT1G71692
AGL15	AT5G13790
AGL17	AT2G22630
AGL25	AT5G10140
AGL69	AT5G65070
AGL68	AT5G65080
AGL27	AT1G77080
AGL70	AT5G65060
AGL31	AT5G65050
AGL32	AT5G23260
TM8	NP_001234105.2
AGL94	AT1G69540
AGL30	AT2G03060
AGL65	AT1G18750
AGL67	AT1G77950
AGL66	AT1G77980
AGL104	AT1G22130
